# Health Tract, No. 101

**Published:** 1862-09

**Authors:** 


HEALTH TRACT, No. 101.

### SORES

Are accidental or spontaneous. They sometimes heal readily; at others, they resist all known remedies, and last for months, years, and even to the close of life. Many persons appear in perfect health, and yet, on inquiry, it will be found that they have had running sores on some part of the body for many years. If a person is in good health, and a sore is made by a bruise, scratch, splinter, or otherwise, it will heal of itself rapidly; but if an invalid, or if of a feeble constitution, the sore will be a long time in healing, and may prove very troublesome. Persons who drink alcoholic liquors have very little healing power, and a slight bruise or abrasion of the skin will be weeks and months in getting well. The men who work about the London breweries drink large quantities of ale and beer every day, and when they get to be forty or fifty years old, the scratch of a pin sometimes becomes fatal; and very slight bruises or cuts are healed with the greatest difficulty.

An abrasion of the skin, where there is but little flesh, as on the "shin," very often becomes a running sore for life, because there is little vitality in the part. A gentleman of wealth, in getting into his carriage, had a slip of the foot, and the forepart of the leg scraped against the iron door-step; it inflamed, spread, ulcerated; mortification took place, and he died. He drank liquor habitually. The healthiest persons should carefully protect any sore on the fore-part of the leg from being rubbed by the clothing. Never allow the "scab" to be picked off; let it fall off of itself.

Sores sometimes come without apparent cause. It is because the blood is "bad," is in a diseased condition, and nature is making an effort to throw it out of the system. The person is apparently well, has a good appetite; tries this thing, that, and the other, but nothing seems to do any good. And nothing will do any good, besides keeping it clean and moist, until nature has relieved herself, until the blood has "run itself" pure; and then the sore heals without any agency. Very often at this turning-point a person happens, on advice, to smear on a little goose-grease, or other inert material, and the sore gets well—not as a consequence, but as a coïncidence—and thereafter, until life's close, goose-grease with that individual becomes a famous remedy, is "good for" sores, and every thing else. The sore in such cases has prevented an attack of fever or other sickness. On the appearance of any sore, it is wise to begin at once, and eat nothing but fruits and coarse bread; keep the body clean, and exercise more freely in the open air, and thus aid nature in working off the offending matters. Life is often lost by healing up a running sore rapidly. It should never be done, unless at the same time the system is kept free by the use of laxative food or medicine. Under such conditions, the most incorrigible scrofulous sores may be soon and safely healed, thus: first wash the sore well, then apply with a brush or soft rag, twice a day, the following: put one ounce of aquafortis into a bowl or saucer; drop in two copper cents; when effervescence ceases, add two ounces of strong vinegar. If it smarts too severely, add a little rain-water.

